# Crise convulsive chez les abuseurs de Tramadol et caféine: à propos de 8 cas et revue de la littérature

**Published:** 2012-10-03

**Authors:** Djibo Douma Maiga, Houdou Seyni, Amadou Sidikou, Alfazazi Azouma

**Affiliations:** 1 Faculté des Sciences de la Santé de Niamey, Niger; 2 Service de Psychiatrie. Département de médecine et spécialités médicales Hôpital national de Niamey, Niger

**Keywords:** Tramadol, caféine, convulsions, addiction, Tramadol, caffeine, convulsions, addiction

## Abstract

Nous rapportons Huit cas de crises convulsives diagnostiquées comme maladie épileptique après ingestion de Tramadol et d'autres substances psychotropes dont la Caféine dans une région ou maladie épileptique et addiction au café sont fréquentes. L'objectif de ce travail était d'informer les praticiens sur le risque de convulsion lié à la consommation du Tramadol seul ou en association avec d'autres psychotropes en s'appuyant sur les données de la littérature. Il s'agissait d'une étude rétrospective et exhaustive de patients vus en consultation ambulatoire pour crise convulsive et consommation de Tramadol et de caféine de janvier à mai 2012. Les données collectées étaient les caractéristiques sociodémographiques et de la consommation de Tramadol. Le diagnostic de crise convulsive a été posé sur les renseignements obtenus à l'anamnèse. Tous les patients ont été soumis à un examen neurologique et aux critères de dépendance du Diagnostic and Statistical Manual of Mental Disorders (DSMIV)-R par rapport à leur consommation de Tramadol. Nous n'avons pas trouvé dans la littérature médicale de cas de consommation concomitante de Tramadol et de Caféine. Les données expérimentales suggèrent une action synergique du Tramadol et de la Caféine sur la douleur et le seuil épileptogène. Nos observations plaident également en faveur d'une synergie d'action de ces deux molécules dans la survenue des crises convulsives. La fréquence des crises convulsives suite à une intoxication par le Tramadol et la caféine est susceptible d'augmenter en Afrique en raison du mésusage croissant de ces substances. Une étude comparative usagers de Tramadol associé à la Caféine et usagers du Tramadol seul devrait permettre d’évaluer le risque.

## Introduction

Le chlorhydrate de Tramadol est une molécule utilisée en tant qu′antalgique. Il fait partie des médicaments antalgiques du palier II de l'OMS. Avec sa diffusion anarchique et croissante à travers les « pharmacies par terre » (nom donné à la vente illicite en point fixe ou en ambulatoire de médicaments pharmaceutiques) et son pouvoir addictogène, son usage tend à devenir exclusivement abusif dans le monde [1, 2].

Outre, la dépendance dont il peut être responsable, le Tramadol peut entrainer consommé seul ou en association à d'autres substances psychoactives diverses complications dont des crises convulsives [3–6]. Cette complication aigue peut être confondue à une maladie épileptique dans la région ouest africaine ou le mésusage du Tramadol et la maladie épileptique sont fréquents.

Pour cette raison, nous rapportons 8 observations de patients vus en consultation pour crises convulsives diagnostiquées comme maladie épileptique après ingestion de substances psychoactives dont le Tramadol. Ces huit observations confrontées aux données de la littérature permettent une discussion sur le diagnostic et sur l'imputabilité des crises convulsives au Tramadol consommé concomitamment à la Caféine.

Le but de ce travail, était d'informer les praticiens, sur la consommation concomitante de Tramadol et de Caféine comme facteur de risque majeur de survenue de crise convulsive et d'installation de maladie épileptique.

## Méthodes

Il s'agissait d'une étude rétrospective et exhaustive de patients vus en consultation ambulatoire pour épilepsie et consommation de Tramadol et Caféine de janvier à mai 2012.

Les données collectées étaient les caractéristiques sociodémographiques (âge, sexe, profession; les antécédents médicaux; le motif de la consultation; la durée de la consommation de Tramadol; la dose consommée précédent la crise convulsive; l′intervalle de temps entre la dernière consommation et la crise convulsive et celui entre la crise et la consultation; la prise en charge au décours de la crise; les autres substances associées à la consommation du Tramadol. Le diagnostic de crise convulsive a été posé à partir des renseignements obtenus à l'anamnèse. Tous les patients ont été soumis à un examen neurologique. Ils ont également été soumis aux critères de dépendance du (Diagnostic and Statistical Manual of Mental Disorders) DSMIV-R par rapport à leur consommation de Tramadol.

## Résultats

Pour tous les patients la consultation intervenait à la demande des parents (père, mère ou frère). Un seul patient (N° 7) a un antécédent médical (drépanocytose AS). Aucun patient n'avait un antécédent de crise épileptique avant la consommation de Tramadol. L'examen neurologique était normal chez tous les patients.

Tous les patients étaient dépendants du Tramadol selon les critères de dépendance de la DSMIV-R. La Caféine était consommée sous forme de liquide de café. La crise convulsive survenait dans les 12 heures qui suivaient la dernière prise de Tramadol. Le motif de la consultation, la durée de la consommation de Tramadol, la dose consommée précédent la crise convulsive, et les substances associées à la consommation du Tramadol sont consignées dans le Tableau 1.

**Tableau 1:** Résumé des observations

N°	Age (ans)	Motif de la demande de la consultation	Durée entre la crise convulsive et la consultation	Dose consommée précédant la crise convulsive	Substances sous forme liquide véhiculant la dose de Tramadol précédant la crise	Autres substances consommée régulièrement	traitement au décours de la crise
1	25	Coma avec hospitalisation aux urgences	2 mois	2400 mg	Caféine (liquide de café)	Thé cigarettes	Hospitalisation aux urgences de l'hôpital
2	21	Délinquance (vols domestiques)	Deux semaines	720 mg	Caféine (liquide de café)	Thé cigarette	
3	17	Consommation abusive de Tramadol	Un jour	1200 mg	Caféine (liquide de café)	Thé cigarette	
4	20	Délire de persécution	Une semaine	1200 mg	Caféine (liquide de café)	Thé cigarette	
6	15	Abandon scolaire et comportement anormal	3 mois	840 mg	Caféine (liquide de café)	cigarette	
7	17	Syndrome amotivationnel avec sentiment de persécution	3 mois	1200 mg	Caféine (liquide de café)	Thé cigarette	
8	14	Abus constaté par les parents	3 semaines	600 mg	Eau		Traitement des douleurs drépanocytaire

Aucun traitement n'a été initié dans les suites immédiates des crises convulsives pour 7 des 8 patients. Pour un patient (N°1) une hospitalisation a été faite aux urgences pour coma post critique.

## Discussion

Les cas que nous rapportons posent la question des facteurs étiologiques d'une première crise convulsive chez des jeunes sans antécédent pathologique consommateurs de Tramadol, de Caféine et d'autres substances psychotropes. Cette question se pose d'autant que les familles se sont présentées avec leur enfants pour épilepsie et divers autres motifs (délinquance, vols, troubles psychotiques, chômage par abandon scolaire ou d'activité professionnelle).

L'entretien a révélé à partir du profil des patients, le mésusage du Tramadol associé à la Caféine. C'est également l'entretien qui a révélé la survenue de crise convulsive chez tous les patients dans les suites immédiates (le même jour) de la consommation concomitante de Tramadol de Caféine.

L'imputabilité du Tramadol dans la survenue des crises a été d'abord évoquée. Des auteurs [3–6] l'ont incriminés dans la survenue de nombreuses complications aigues dont les crises convulsives dans des situations d'addiction ou d'intoxication accidentelle. Pour Petramfar [4] les quantités ingérées responsables de crises convulsives variaient entre 50 à 1500 mg. Nos patients consommaient des quantités qui variaient entre 880 et 1200 mg par jour. Ils étaient tous dépendants du tramadol, le phénomène de tolérance pourrait expliquer les quantités ingérées. Le mécanisme par lequel le Tramadol entraine des crises convulsives est encore mal connu.

Le Tramadol agirait comme antagoniste de l'adénosine au niveau des récepteurs du cerveau [7–10]. Cette substance a une propriété anticonvulsivante prouvée par l'efficacité des traitements qui augmentent sa teneur au niveau du cerveau et la survenue de crises convulsives liées à son déficit cérébral [4, 8]. Le Tramadol agirait également seulement à forte dose sur le seuil épileptogène en inhibant le récepteur de la GABA A [10].

La survenue de crise convulsive en cas d'ingestion de Tramadol n'est pas systématique, elle est dose dépendante et ferait intervenir également d'autres facteurs [10]. Les interactions médicamenteuses ont été incriminées de même que la consommation de plusieurs substances psychoactives [10–13].

Le liquide de café (Caféine) constituait le véhicule systématique des comprimés de Tramadol chez 7 des 8 patients. Moussa [14] rapportait que 91,5% des 46 dépendants du Tramadol utilisaient le liquide de café comme véhicule des comprimés de Tramadol. Or il est connu que Les méthylxanthines (Caféine, théophylline, théobromine, paraxanthine) peuvent être responsables de crises convulsives [12–15].

Ces substances ont également des activités antagonistes de celle de l'adénosine par plusieurs mécanismes dont: inhibition du récepteur A1 de l'adénosine et du flux sanguin cérébral; abaissement du seuil épileptogène; inhibition de la 5 nucléotidase et diminution de la production d'adénosine endogène; inhibition de pyridoxine kinase (enzyme nécessaire à la synthèse de la GABAA); augmentation de la GMP cyclique; inhibition directe du récepteur de la GABAA.

Le mécanisme par lequel les methylxathines entrainent les convulsions chez l’épileptique dépendent du mode d'usage et de la dose [12, 15]. Un usage aigu et à forte dose chez un non consommateur abaisserait le seuil épileptogène, facilitant l’émergence d'une crise d’épilepsie généralisée alors qu'un usage chronique à dose modérée bloquerait les récepteurs A2a conduisant ainsi à une élévation du seuil épileptogène.

L'ingestion concomitante de doses importantes de Tramadol et Caféine a déclenché une crise convulsive chez tous nos patients qui n’étaient usagers réguliers des deux substances.

L′administration concomitante de Caféine et de Tramadol a été démontrée comme agissant en synergie contre la douleur en expérimentation animale [16]. Une autre étude expérimentale récente indique que la Caféine n'altérait pas le seuil épileptogène au pentylénetetrazole mais diminue le seuil de déclenchement de crise convulsive par électrochoc chez le rat [17], alors que le Tramadol réduit le seuil épileptogène au pentylénetetrazole, mais élève le seuil de déclenchement de crise convulsive par électrochoc. Il faut noter qu'en plus de la Caféine et du Tramadol, 7 des 8 de nos patients fumaient la cigarette, qui est un autre facteur de risque de convulsion et d’épilepsie [12].

Les hypothèses explicatives ci-dessus ne permettent pas imputer formellement les crises convulsives à telle ou telle substances (Tramadol, café, cigarette). Elles plaident en faveur d'une synergie d'action dans la genèse des crises.

Une question reste celle de l'avenir des telles crises, dans un tel contexte. La poursuite de la consommation de ces substances pourrait augmenter le risque de récurrence des crises convulsives voir d'installation d'une maladie épileptique. Il est connu que les crises convulsives survenant après un désordre métabolique ou toxique ont un faible risque de devenir récurrentes (≤ 3%) et celles provoquées par des troubles entrainant une lésion permanente du cerveau ont un risque plus élevé de devenir récurrentes (≤ 10%) [18].

Une seconde question reste la conduite immédiate à tenir devant de tel patient. Bien sûr qu'il faut créer les conditions (évaluation, mise en place d'entretien motivationnel et traitement des comorbidités éventuelles etc.) qui vont favoriser l'abstinence, mais faut il aussi mettre en place un traitement antiépileptique et les mesures de vie restrictives qu'impose le principe de réduction de risque lié à d’éventuelles crises futures?

La réponse à cette question pourrait venir aussi des résultats d'explorations complémentaires (EEG, IRM, le dosage sanguin et urinaires des molécules). Ce qui n'est pas toujours et partout disponible en Afrique.

Ces huit observations présentent l′avantage de mettre en relief non pas tant le raisonnement médical conduisant au diagnostic qui devrait être basé sur: L'usage illicite en raison de la disponibilité et de l'accessibilité facile des ces produits; La notion d′abus et de dépendance; La notion de poly consommation (tabac, café, autres psychotropes etc.); La proximité temporelle entre la consommation et la survenue de la crise convulsive. Mais la difficulté d'obtenir des informations objectives (dosage sanguin des substances, IRM, EEG etc.) pendant les périodes critiques ou postcritiques immédiates pour affirmer l'imputabilité formelle de ces substances dans la survenue des crises convulsives.

## Conclusion

La fréquence des crises convulsives par intoxications par de Tramadol et de Caféine est susceptible d'augmenter, car il s'agit de psychotropes dont les usages sont abusifs en l'Afrique de l'Ouest. Ce type d'association (poly toxicomanie) devra être connu du tout clinicien du fait de la potentielle augmentation de risque d'installation de maladie d’épileptique dont les représentations sociales et culturelles sont encore redoutables.
